# *RepPop*: a database for repetitive elements in *Populus trichocarpa*

**DOI:** 10.1186/1471-2164-10-14

**Published:** 2009-01-09

**Authors:** Fengfeng Zhou, Ying Xu

**Affiliations:** 1 Computational Systems Biology Laboratory, Department of Biochemistry and Molecular Biology, and Institute of Bioinformatics, University of Georgia, Athens, GA 30602, USA; 2 BioEnergy Science Center, Tennessee, USA

## Abstract

**Background:**

*Populus trichocarpa *is the first tree genome to be completed, and its whole genome is currently being assembled. No functional annotation about the repetitive elements in the *Populus trichocarpa *genome is currently available.

**Results:**

We predicted 9,623 repetitive elements in the *Populus trichocarpa *genome, and assigned functions to 3,075 of them (31.95%). The 9,623 repetitive elements cover ~40% of the current (partially) assembled genome. Among the 9,623 repetitive elements, 668 have copies only in the contigs that have not been assigned to one of the 19 chromosome while the rest all have copies in the partially assembled chromosomes.

**Conclusion:**

All the predicted data are organized into an easy-to-use web-browsable database, *RepPop*. Various search capabilities are provided against the *RepPop *database. A Wiki system has been set up to facilitate functional annotation and curation of the repetitive elements by a community rather than just the database developer. The database *RepPop *will facilitate the assembling and functional characterization of the *Populus trichocarpa *genome.

## Background

The *Poplar *was selected to be the first tree genome to be sequenced, mainly because of its extraordinarily rapid growth rate and its relatively compact genome size (450–500 Mbps [1,2]). Biofuels are produced mainly through two sources, i.e. crops high in sugar or cellulose, e.g. sugar canes [3] and plants [4], and plants high in vegetable oils like soybean[5]. The *Populus trichocarpa *genome's rapid growth coupled with the high content of lignocelluloses has made it one of the model systems for the new generation of biofuels [4]. The current assembly of the *Poplar *genome was released in June 2004, and its total length is ~485 Mbps. The assembled 19 chromosomes with 7.66% gaps count for 63.41% of the whole genome. Further efforts are still needed to close the gaps in the sequenced chromosomes.

Repetitive elements represent a significant fraction of eukaryotic genomes and they could occupy as high as 80% of some land-plant genomes like wheat [6] and as low as 10–35% for *Arabidopsis thaliana *[7] and rice [8]. There are three main classes of repetitive elements, namely, local repeats (tandem and satellite repeats) [9], interspersed repeats (transposons) and segmental duplications (duplicated genomic segments). Among them, transposable elements are the most extensively studied repetitive elements, and they can be classified as retrotransposons or DNA transposons based on whether they are transposed through the RNA or DNA intermediates [10]. Both interspersed repeats [11-16] and other duplicated elements [17] may induce homologous recombinations and insertions/deletions in the host genome, which may introduce great difficulties to the correct assembly of the repetitive regions in the host genome.

Typically repetitive elements have been identified in a genome using two approaches: (1) identification of homologous sequences to known repetitive elements [18], and (2) identification of repeats based on self-comparison a given genome and clustering them into families [19-21]. The first approach requires manually curated repetitive elements, which may not be feasible for newly sequenced genomes, though it can identify the precise boundaries of repetitive elements, even for the embedded partial copies. The second approach identifies repetitive elements in a *de novo *fashion, though it may require additional manual curations for the boundaries of the predicted elements.

## Construction and content

### Data resources

The current assembly of the *Populus trichocarpa *genome was released in June 2004 as version 1.1, which consists of 22,012 nucleotide sequences, covering large pieces of the 19 chromosomes and some unassembled short contigs, and the total length is 485,510,911 bps. This data was downloaded from the web site of *Populus trichocarpa *genome sequencing project [22].

We downloaded four of the most comprehensive databases of repetitive elements in eukaryotes, RepBase [23] version 12.05 (release of July 13, 2007), TREP [24] version 10 (release of July 2008), RetrOryza [25] and AtRepBase [26], for homology search. We also downloaded the databases RDP [27] and Rfam [28], and RNA genes in the rice RAP-DB database [29]. The NCBI database NT [30] containing all the non-redundant protein sequences was also downloaded for homology search.

### Identification of repetitive elements

Due to the very large computer memory requirement by many repeat identification programs [19-21], we implemented our *RepPop *database and associated tools on a 64-bit Linux operating system with 32 GB memory. The repetitive elements with at least 2 copies in the *Poplar *genome were identified using *RepeatScout *[19]. We then removed any repetitive elements predicted to be low complexity regions using program *NSEG *[31] and tandem repeats using program *TRF *[32]. All the programs were run using the default parameters.

**Figure 1:**
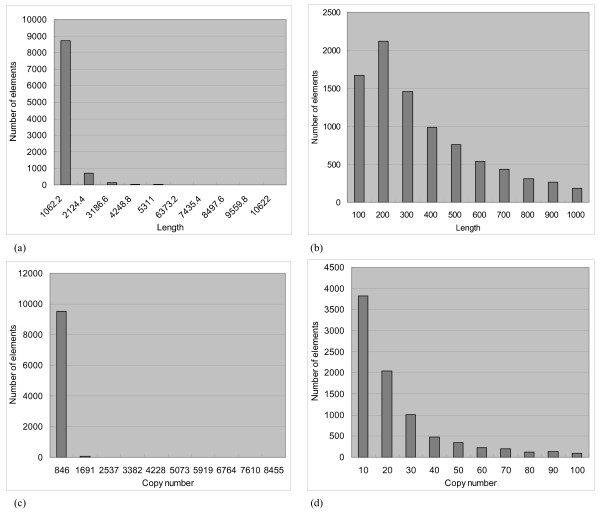
**Basic information of the identified 9,623 repetitive elements**. Distributions of (a) lengths and (c) copy numbers of repetitive elements in the *Populus trichocarpa *genome. The two distributions within a shorter range are in (b) and (d).

Totally 9,623 repetitive elements were identified, covering 194.00 Mbps (~40%) of the *Poplar *genome. The distributions of copy numbers and lengths of these repetitive elements are given in Figure 1. Most of the repetitive elements are short and of low copy numbers.

### Annotation procedure

We first identified the homologous regions of the 9,623 repetitive elements in the databases *RepBase *[23], TREP [24], RetrOryza [25] and AtRepBase [26] using the NCBI Blast [30] with *E-value *cutoff e-5. One region might match two homologous elements in the database. We then removed the redundant annotations by keeping only the region with the lowest *E-value *for the overlapping regions. A total of 226 homologous regions were identified.

We then predicted 30 tRNA genes using the program *tRNAscan-SE *with default parameters [33]. 8 and 40 homologous regions to the RNA genes in databases *RDP *[27] and *RAP-DB *[29] were identified using the NCBI Blast [30] with *E-value *cutoff e-5 after removing the redundancy like above. No homologous regions were identified based on the RNA profiles of *Rfam *[28] using the program *infernal *[19] with default parameters.

2,720 homologous regions to sequences in the database NT [30] were identified using NCBI Blast [30] with *E-value *cutoff e-5, and annotated as having the functions of the best matched homologous proteins.

## Utility and discussion

**Figure 2:**
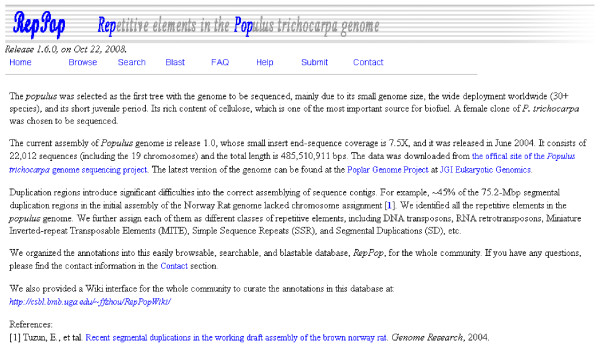
**The main web page of database *RepPop***.

We organized the 9,623 predicted repetitive elements and their annotations into an easy-to-use web-browsable database system, *RepPop *[34] (Figure 2). *RepPop *is and will continue to be under continuing update on quarterly basis for annotation and curation of these repetitive elements. The composition of the *RepPop *database can be found in Table 1. We provided a Wiki interface for the whole community to help curate the annotations [35].

**Table 1:** Basic knowledge of the *RepPop *database

**Annotations**	**Number**	**Number%**	**Length (bps)**	**Length%**
Transposons	161	1.67	21,044,639	4.33

RNA genes	15	0.16	36,051	0.01

Protein-coding genes	2,983	31.00	157,586,923	32.46

### Data browsing

**Figure 3:**
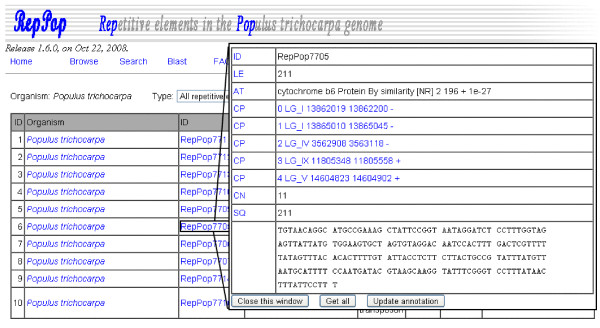
**The browsing interface of database *RepPop***. All 9,623 predicted repetitive elements in the *Populus trichocarpa *genome. The user may click the entries in the column REName to retrieve detailed annotation of each repetitive element. A detailed description of the plant *Populus trichocarpa *could be found in a popup window by clicking on *Populus trichocarpa*.

A user may browse all the 9,623 repetitive elements in the browsing interface of *RepPop*, as shown in Figure 3. The detailed annotation of each repetitive element can be retrieved using a popup window by clicking the corresponding entry under REName. Some repetitive elements have as high as 8,455 copies (RepPop694) in the *Populus trichocarpa *genome. We believe that it is not necessary to list the information of all the copies for such repetitive elements. So the browsing interface lists the information of at most 5 copies for each repetitive element as the default. The user can get the additional information, if needed, of all the copies by clicking the button "Get all". The user could also choose to browse only one of the following types of repetitive elements, namely, transposons, RNA genes, protein coding genes, and repetitive elements with no annotations, by clicking the corresponding entry in the drop-down menu.

### Data search with key words

**Figure 4:**
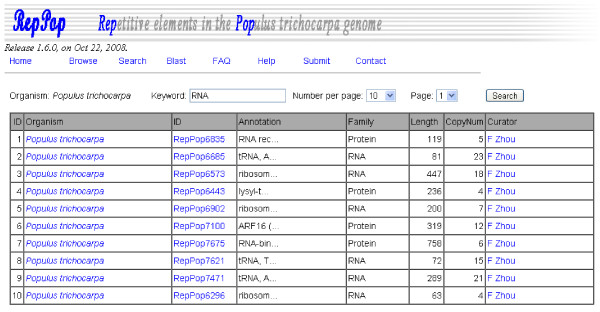
**The searching interface of database *RepPop***. A user can search for repetitive elements with keywords through the keyword search interface.

The keyword search interface of *RepPop *makes it possible for a user to find items interesting to the user using a few keywords, as shown in Figure 4. Besides the typical keyword matching in the annotations, *RepPop *also provides the flexibility to support search for items within a specified range in terms of, say, elements with certain lengths or with certain copy numbers. For example, a pattern like "Length:Min-Max" as part of a keyword search can be used to find repetitive elements whose lengths are between the specified parameters Min and Max, whose default values are 0 and 10,622 if such range information is not specified. 10,622 is the maximum length of repetitive elements. Another example is including "Length>100" as part of a keyword search to find repetitive elements with at least 100 bps; and similarly "Length<500" is used to find elements with at most 500 bps. Similar range specifications are also available for specifying other quantities, say, "CopyNum:Min-Max" used for finding repetitive elements with copy numbers between specified parameters Min and Max. Tips for keyword searches are available in the Help box of *RepPop*.

### Sequence homology search

**Figure 5:**
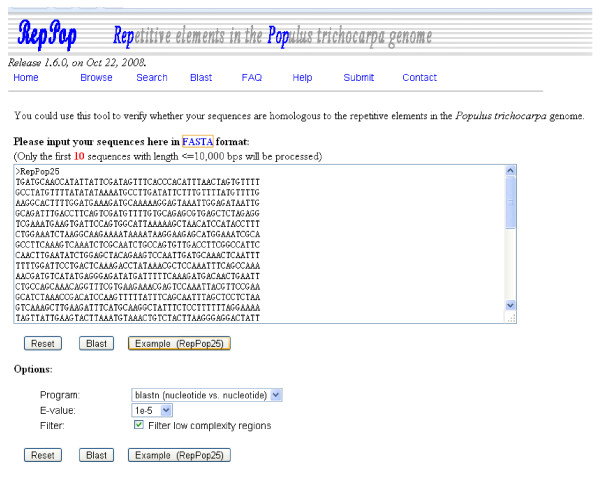
**The blasting interface of database *RepPop***. Searches for the homologous regions for user-specified DNA or protein sequences in *RepPop*.

An interface is provided to facilitate Blast search, using the NCBI Blast, against the 9,623 repetitive elements. Through this interface (Figure 5), a user can simultaneously specify up to 10 nucleotide or protein sequences of no more than 10 kbps for homology search. A query example is provided for using this interface and can be found and used by clicking the button "Example (RepPop25)".

### User input to RepPop

**Figure 6:**
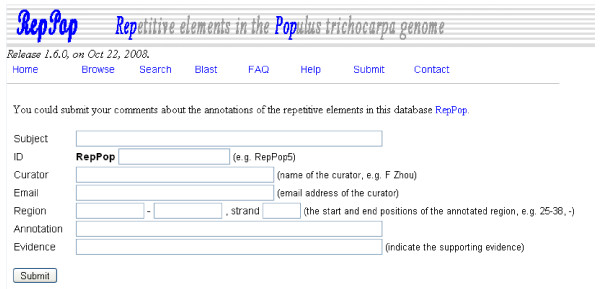
**The submision interface of database *RepPop***. A user of *RepPop *may submit his/her annotations on a specific repetitive element with supporting evidence through this interface.

**Figure 7:**
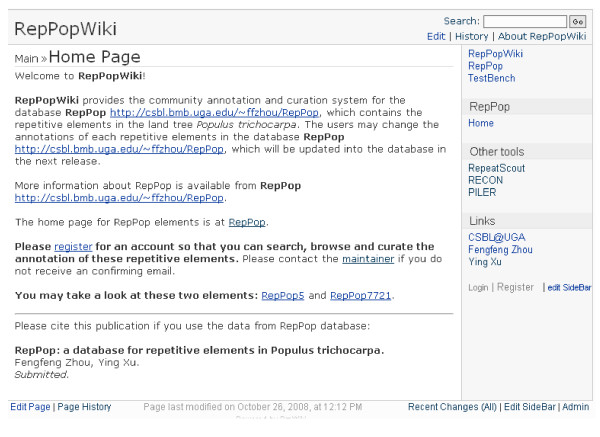
**The Wiki interface of database *RepPop***. A user can revise the annotation of a specific repetitive element through this interface.

We were able to assign functions for 3,075 of the 9,623 repetitive elements, based on homology search against the NT database, leaving 6,548 (~68.05%) repetitive elements functionally unassigned. We have designed the *RepPop *interface in such a way that a user can submit his/her own functional annotations of any repetitive elements in *RepPop *through the Submission interface, as shown in Figure 6. We have provided a Wiki web site for the general user community to directly annotate and curate the assigned functions of repetitive elements and to keep track of updates of annotations of each repetitive element, using PmWiki [36]. The developer of the *RepPop *database has the ultimate right to keep, revise or delete a particular contribution made by a user, which will be done on regular (say, monthly) basis, based on the input provided by the users through PmWiki. Figure 7 shows a screen shot of using this feature. The user needs to register to open an account through the provided link in the right sidebar of main interface of RepPopWiki [35] before being able to add and revise the annotations for selected elements.

### Manual input and other useful information

A Help interface is provided to help the users to get familiar with how to use *RepPop*. A detailed description of using various interfaces of *RepPop *can be found on this page. A collection of comprehensive databases of repetitive elements and computational programs for identifying such elements is provided in this Help interface, a user of which may be interested in identifying repetitive elements in other genomes. A list of Frequently Asked Questions (FAQs) is included in the FAQ interface.

### Comparison with other databases of plant repetitive elements

There are quite a few databases focusing on the repetitive elements in plants. RetrOryza [25] collects 242 families of LTR retrotransposons in the rice genome, AtRepBase [26] provides the browsing and blasting interfaces for the 63 well annotated repetitive elements in the *Arabidopsis *genome and TREP [24] represents a community joint effort to collect and annotate the repetitive elements in the *Triticeae *genomes. All above three databases collect a limited number of repetitive elements with well curated annotations in one or a few closely related organisms. Our database, *RepPop*, computationally identified all the families of repetitive elements and tried to annotate them using sequence mapping. We have classified them as RNA, transposon and unknown genes, which is similar to the classification system of TREP.

## Conclusion

*RepPop *is a database currently consisting of all the 9,623 predicted repetitive elements in the *Populus trichocarpa *genome along with functional annotations for some of them. Various search capabilities are provided in support of using this database by a large community of users. One unique feature of the database is that it allows users to add their annotations and curations to selected repetitive elements in a fashion similar to Wikipedia, which should help to rapidly increase the amount of information stored in this database.

## Future perspectives

More efforts are being put into manual curations to provide more accurate annotations of the predicted repetitive elements, especially for the chimeric ones. Curations from other researchers, including users, are encouraged, as discussed above, through the web site of *RepPop*.

## Availability and requirement

Project name: The repetitive elements in *Populus trichocarpa *genome.

Project home page: .

Operating system(s): Platform independent.

Programming languages: PHP.

License: Not required.

Any restrictions to use by non-academics: None.

## Abbreviations

*RepPop*: Repetitive elements in the *Populus trichocarpa *genome; IS: Insertion Sequence; LTR: Long Terminal Repeat.

## Authors' contributions

FZ conceived the project, performed the identification and annotation of the data, and wrote the manuscript. YX wrote and polished the manuscript, and served as the principle investigator of the project. All authors have read and approved the final submitted version of this manuscript.
